# *Eucommia* ulmoides polysaccharide modified nano-selenium effectively alleviated DSS-induced colitis through enhancing intestinal mucosal barrier function and antioxidant capacity

**DOI:** 10.1186/s12951-023-01965-5

**Published:** 2023-07-12

**Authors:** Ruihua Ye, Qingyun Guo, Jiaqiang Huang, Zixu Wang, Yaoxing Chen, Yulan Dong

**Affiliations:** 1 College of Veterinary Medicine, China Agricultural University, Beijing, 100193 China; 2 Qingyun Guo,Milu conservation research unit, Beijing Milu Ecological Research Center, Beijing, 100076 China; 3 Key Laboratory of Precision Nutrition and Food Quality, Department of Nutrition and Health, Ministry of Education, China Agricultural University, Beijing, 100193 China; 4 Beijing Advanced Innovation Center for Food Nutrition and Human Health, Department of Nutrition and Health, China Agricultural University, Beijing, 100193 China

**Keywords:** IBD, *Eucommia* ulmoides polysaccharide, Nano-selenium particle, Colitis, Intestinal mucosal barrier, Gut microbiota

## Abstract

Ulcerative colitis (UC) is currently the most common inflammatory bowel disease (IBD). Due to its diverse and complex causes, there is no cure at present, and researchers are constantly exploring new therapies. In recent years, nano-selenium particle(SeNP) has attracted wide attention due to excellent biological activities. Therefore, in this study, for the first time, we used a natural polysaccharide, *Eucommia* ulmoides polysaccharide (EUP), modified SeNP to get EUP-SeNP with a size of about 170 nm, and its effect on 3% dextran sulphate sodium (DSS) induced colitis was explored. Our results showed that colon intestinal histology, intestinal mucosal barrier, inflammatory cytokines and intestinal microbiome composition were changed after EUP-SeNP treatment in colitis mice. Specifically, it was also shown that oral treatment of EUP-SeNP could relieve the degree of DSS-induced colitis in mice by restoring weight loss, reducing disease activity index (DAI), enhancing colon antioxidant capacity and regulating intestinal microbiome composition. In addition, we verified the mechanism in intestinal epithelial cell lines, showing that EUP-SeNP inhibited LPS-induced activation of the TRL-4/NF-κB signaling pathway in intestinal epithelial cell lines. To some extend, our study provides therapeutic reference for the treatment of IBD.

## Introduction

Ulcerative colitis (UC) is a chronic, non-specific inflammation of the colon and rectum, mainly manifested as pathological intestinal mucosal injury, ulceration, contraction of the intestine, diarrhea, blood in the stool [1, 2]. It is worth noting that UC is the result of a combination of factors [3, 4], such as genetic factors, environmental factors, changes in gut microbiota, immune system diseases, and oxidative damage. 5-aminosalicylic acid, corticosteroids, immunomodulators, and biologics (such as adalimumab) are widely used in the clinical treatment of UC [2, 5]. However, these agents are thought to be expensive and can even cause some undesirable side effects [6, 7]. Therefore, it is urgent to find a new substance to treat UC.

Selenium(Se), an essential trace element, was thought to be toxic when it was first discovered, and its biological role was gradually discovered [8]. It has strong anti-inflammatory, antioxidant and anticancer biological activities [9]. Dietary Se supplementation can effectively alleviate the degree of colitis and reduce the occurrence of cancer, such as colon cancer and prostaglandin cancer [10, 11]. Se can be divided into organic and inorganic Se. The bioavailability of organic Se is higher than that of inorganic Se. SeNP is a new Se preparation, it has been widely used as drug carriers due to its small size, large specific surface area and unique physical and chemical properties [12–14]. Compared with organic and inorganic Se compounds, SeNP has higher antioxidant activity, better bioavailability and lower toxicity [13, 15]. However, SeNPs are very unstable in the liquid phase and are easy to aggregate into gray or black Se with large particle size, thus losing the bioavailability and bioactivity of SeNP. Therefore, suitable stabilizer is needed to improve its stability [16]. It has been reported that various some biological macromolecules such as proteins, polysaccharides, polyphenols can modify SeNP, and the modified SeNP has been found to have greater biological activity [17]. Naturally bioactive polysaccharides, which are rich in hydrophilic groups such as hydroxyl groups, have attracted increasing attention and are considered as ideal templates for stabilizing SeNP [18–20]. Chondroitin sulphate SeNP has a protective effect on Alzheimer’s disease mice [21], SeNP covered by polysaccharide - protein complex can promote the growth of tilapia [22], Astragalus polysaccharide SeNP can significantly inhibit the proliferation of HepG2 cells and induce morphological changes of HepG2 cells, and finally trigger the apoptosis of HepG2 cells through mitochondrial pathway [18]. E. ulmoides is a species of euzhongaceae with medicinal and industrial value. It is a dioecious woody plant [23]. EUP are the general term of sugars extracted from the leaves and roots of Euzhongaceae. Previous studies have shown that EUP has anti-inflammatory, antioxidant and immunomodulatory functions [24, 25].

In addation, NF-κB is usually related to inflammatory and in the cytoplasm it is inactive but phosphorylated in response to extracellular factor stimulation. Phosphorylated NF-κB can enter the nucleus to bind to target genes and regulate transcription [26]. TLR-4 is a major inflammatory inducer among members of the TLR family, and TLR4-mediated inflammatory related intestinal damage can accelerate the development of UC [27]. Inhibition of the TRL-4/NF-κB signaling pathway is usually the first choice for anti-inflammatory effects.

In this study, we used EUP as a surface modifier to modify SeNP to obtain a stable SeNP with anti-inflammatory and antioxidant functions, and then explored its effect on DSS-induced colitis. Finally, we investigated the effect of EUP-SeNP on the LPS-activated TRL-4/NF-κB signaling pathway in intestinal epithelial cell lines so as to provide a reference for the treatment of UC.

## Materials and methods

### The preparation of EUP - SeNP

SeNP was prepared by reacting sodium selenite (Aladdin Industrial Corporation, Shanghai, China) with ascorbic acid (Solarbio, Beijing, China) in a certain proportion. In brief, 10 mL sodium selenite (20 mM) was added to 10 mL ascorbic acid (80 mM) for mixing and stirring for 1 h under dark environment, and then *Eucommia* ulmoides polysaccharide (Tianrui Biology, Xian, China) (0.32, 0.64, 1.28, 1.71, 2.13 mg/mL) was added to stir for 12 h to obtain EUP-SeNP solution. Excess sodium selite and ascorbate were subsequently removed by dialysis with Mili-Q water in regenerated cellulose tubes (Mw cutoff value 5000) until the Se regenerated cellulose tubes were undetectable by inductively coupled plasma emission spectrometry (ICP-OES) in external solution. After dialysis, some of the reaction products were lyophilized. The remaining liquid is stored at 4 ℃.

### Characterization of EUP-SeNP

The Se content in EUP-SeNP was quantitated by ICP-OES on an ICP spectrometer at a specific wavelength of 196.08 nm. The Fourier transform infrared (FTIR) spectra of EUP and SeNP and EUP-SeNP were determined. Briefly, 5 mg of each sample was uniformly mixed with dry KBr solid powder. The powder was sufficiently ground with a mortar and pressed by a vacuum tablet machine. The infrared absorption spectra of these samples were determined on a Nicolet 6700 FTIR (Thermoelectric (Shanghai) Technology Instrument Company, China). The EUP-SeNP were characterized by transmission electron microscope and the energy dispersive X ray spectrometer (TEM-EDX) (TECNAI G220; FET company, USA). A small amount of the sample was placed on the membrane surface of the copper mesh used for electron microscopy. Then, the copper mesh was dried naturally in the oven to avoid the contamination of dust and other impurities. The dried copper mesh was observed with a TEM, and the elemental composition of the composite particles in the target area was observed using a spot sweep method together with an EDX. Particle size was determined by Zetasizer Nano ZS (ZS90; Malvern Instrument Co., Ltd., UK). Vitro antioxidant capacity was evaluated using 1,1-diphenyl-2-picrylhydrazyl(DDPH), hydroxyl radical(·OH), and 2, 2’-azino-bis(3-ethylbenzothiazoline-6-sulfonic acid)(ABTS) free radical scavenging kit. (Nanjing Jiancheng Bioengineering Institute, China)

### Animals and experimental design

A total of 60 6–8 week old male C57BL/6JNifdc mice (20-21 g) were purchased from Vital River Laboratory Animal Technology Company (Beijing, China). All animals were raised in a controlled environment: relative humidity was 40 ± 10% and temperature was 20 ± 5 ℃, with a 12/12 h light–dark cycle. After 1 week of adaptation, the mice were randomly divided into 6 groups, namely control group, 3%DSS group, 3%DSS + EUP group, 3%DSS + SeNP group, 3%DSS + EUP-SeNP group and 3%DSS + selenite group, each with 10 mice. Except the control group, the other groups were given 3% w/v DSS drinking water for 7 consecutive days, and the control group was given drinking water without DSS. From the 8th day, in the treatment group, 200 µL of the drug was intragastric for 5 days, and the selenium content in the EUP-SeNP treatment group was 0.5 ppm. The control group and DSS group were given the same amount of PBS. Body weight and disease activity index (DAI) scores of mice in each group were observed and recorded daily. The length of the colon was measured when the mice were sacrificed. All animal procedures were approved by the China Agricultural University Institutional Animal Care and Use Committee (AW80212202-2-1).

### Evaluation of colitis

After administration of DSS drinking water, the disease activity index score was recorded every day. The DAI score is a composite score of weight change, stool consistency and stool bleeding. Colon tissues were removed and fixed with 4% paraformaldehyde. The colons were cut into 5 μm sections and stained with hematoxylin & eosin (H&E) and Alcian blue-periodic acid-Schiff (AB-PAS) according to standard procedures. (Solarbio, Beijing, China) Histopathological score is a comprehensive measure of inflammatory cell infiltration and changes in tissue damage. The changes in goblet cells in colon tissue were observed by AB-PAS staining. Detailed scoring criteria for DAI and histology are based on previous articles [28, 29].

### ELISA and measurement of oxidative stress

Proteins were extracted from colon tissues with physiological saline, then detected with a BCA protein analysis kit (CoWin Biotech Co., Inc., Beijing, China). IL-6, IL-10, IL-12, IL-17, IL-1β, TNF-ɑ (Laibotairui Tech Co., Ltd., Beijing, China), were determined by an ELISA kit. The results were normalized to the protein concentration of each sample. A microplate reader (BioTek Co., Ltd., Beijing, China) was used for detection at 450 nm. Indicators of intestinal antioxidant capacity: Total antioxidant capacity (T-AOC), Superoxide Dismutase (SOD), Catalase (CAT), glutathione peroxidase(GPX), Malondialdehyde(MDA), Glutathione/oxidized Glutathione(GSH/GSSG), myeloperoxidase(MPO) levels were determined by using commercial kits according to the manufacturer’s instructions (Nanjing Jiancheng Bioengineering Institute, China).

### Western blotting

Proteins extracted from the colon tissue were separated by 8–12% SDS-PAGE after the concentration was detected by the BCA protein assay kit (CW0014S, CoWin Biotech Co., Inc., Beijing, China). They were then transferred to 0.2 μm polyvinylidene fluoride membranes (Merck KGaA Co., Ltd., Darmstadt, Germany). Membranes were blocked with 5% skim milk for 1.5 h. After blocking, the membranes were incubated with different primary antibodies at 4 ℃ overnight. Claudin 1 (1/3000, Abcam Co., Inc., Cambridge, UK), Claudin 3 (1/1000, Abcam Co., Inc., Cambridge, UK), Occludin (1/1000, Abcam Co., Inc., Cambridge, UK), ZO-1 (1/1000, Abcam Co., Inc., Cambridge, UK), TRL-4(1/1000, Proteintech Co., Wuhan, China), IKB(1/1000, Abmart Co., Inc, Shanghai, China), p65(1/5000, Abmart Co., Inc, Shanghai, China), p-IKB(1/1000, Abcam Co., Inc., Cambridge, UK), pp65(1/1000Abcam Co., Inc., Cambridge, UK), β-actin(1/1000, LABLEAD Biotech Co., Ltd., Beijing, China). Then, the membranes were washed with Trisbuffered saline Tween (TBST) and then incubated with horseradish peroxidase-conjugated goat anti-mouse IgG, or goat anti-rabbit IgG (CoWin Biotech Co., Inc., Beijing, China) for 1.5 h. The membranes were imaged with a Tanon 5200 imaging system. (Tanon Science & Technology Co., Ltd., Shanghai, China)

### Immunohistochemistry

Colon tissue sections were dewaxed with xylene and hydrated in gradient ethanol. Then, the antigen was repaired with 0.01 M sodium citrate buffer. Tissue sections were washed with phosphate buffer (PBS, pH7.0) 3 times for 5 min each time. Endogenous peroxidase activity was blocked by 3% hydrogen peroxide for 30 min, and non-specific staining was blocked by 5% goat serum for 30 min. Tissue sections were incubated with Ki67 antibody (1/200) and anti-MUC2 (1/2000, Abcam Co., Inc., Cambridge, UK) at 4 ℃ overnight. On the 2 day, after being washed with PBS, they were incubated with biotin-conjugated goat anti-rabbit IgG (CoWin Biotech Co., Inc., Beijing, China) for 2 h and then incubated with horseradish peroxidase (HRP)-streptavidin (CoWin Biotech Co., Inc., Beijing, China) for 2 h. DAB chromogenic reagent kit (Zhongshan Jinqiao Biotech Co., Ltd., Beijing, China) was used for chromogenic reagent detection. Hematoxylin was used for nuclear re-staining. The primary antibody of the negative control group was replaced with PBS. Images were captured using a microscope (Nanjing Jiangnan Novel Optics Co., Ltd., Nanking, China).

### TUNEL

Through the one step TUNEL apoptosis assay kit (LABLEAD Biotech Co., Ltd., Beijing, China), terminal deoxynucleotidyl transferase dUTP nick end labeling (TUNEL) assay was used to detect the apoptotic level of colon, IEC-6 and Caco-2. The images were taken with upright DP72 microscope. (Olympus Co., Inc., Tokyo, Japan)

### Microbiota analysis

The contents of the mouse colon were collected and stored in liquid nitrogen. An MN Nucleo Spin 96 Soi DNA extraction kit (MACHEREY-NAGEL GmbH & Co. KG, Duren, Germany) was used to extract total bacterial DNA from the sample. The primers were designed according to the conserved region of micro-organism V3 + V4. The primers were used for PCR amplification, and the products were purified, quantified and homogenized to form a sequencing library. The constructed library was first subjected to quality inspection, and the qualified library was sequenced with Novaseq 6000 (Illumina, Co., Inc., San Diego, CA, USA). Sequence similarity greater than 97% was classified as an operational taxonomic unit (OTU). The OTU composition of different samples was analyzed using principal component analysis (PCA), principal coordinates analysis (PCoA) and non-metric multidimensional scaling (NMDS) based on Bray Curtis analysis. Line discriminant analysis (LDA) effect size, also known as LEfSe, was used to analyze the significance of differences between groups from the phylum to genus level. LEfSe analysis required an LDA score > 4.

### Evaluate the security of EUP-SeNP

We evaluated short-term and long-term toxicity by orally administering EUP-SeNP to mice for 7 days and 30 days, respectively, and then detected Aspartate transaminase (AST) and Alanine transaminase (ALT) of serum. (Nanjing Jiancheng Bioengineering Institute, China) Finally, H&E staining was performed on heart, liver, spleen, lung, kidney and colon.

### Cell viability analysis and cellular uptake of EUP-SeNP

IEC-6 and Caco-2 were cultured in DMEM medium supplemented with 10% FBS, 100 U/mL of penicillin, and 100 µg/mL of streptomycin at 37 °C with 5% CO2 in a humidified atmosphere. Cells were incubated with 50 µg/mL LPS for 36 h, and EUP-SeNP was incubated with 10 µg/mL, 20 µg/mL and 40 µg/mL for pretreatment and post-treatment for 12 h, respectively.

IEC-6 was incubated into a 96-well plate, and then incubated for 24 and 48 h by adding different concentrations of EUP-SeNP. Next, the medium was discarded and washed three times with PBS. Finally, 100 µL PBS and 10 µL CCK8 (Solarbio, Beijing, China) were added to each well and incubated for one hour at 37℃, the optical density was measured at a wavelength of 570 nm. IEC-6 was seeded into a 6-well plate, labeled with fluorescence EUP-SeNP using coumarin-6 as a fluorescence probe, cell nucleus were stained with DAPI (Solarbio, Beijing, China) and cell membranes were stained with Dil (Beyotime Institute of Biotechnology, Jiangsu, China). Cell uptake was observed within 1 h, 2 h, and 4 h, and photographs were taken with fluorescence microscope.

### Statistical analyses

Data analysis was performed with GraphPad Prism (version 8.0.2 for Windows, GraphPad). Data are expressed as the means ± standard errors of the mean (SEM). All comparisons of variant parameters between groups were made with one-way analysis of variance, with statistical significance as follows: * p < 0.05, ** p < 0.01, *** p < 0.001, **** p < 0.0001, #P < 0.05, ##P < 0.01, ###P < 0.001, ####P < 0.0001. “*” represents the significance between the control group and the DSS model group; “#” represents the significance between the DSS model group and the treatment group.

## Results

### Preparation and characterization of EUP-SeNP

**Fig. 1:**
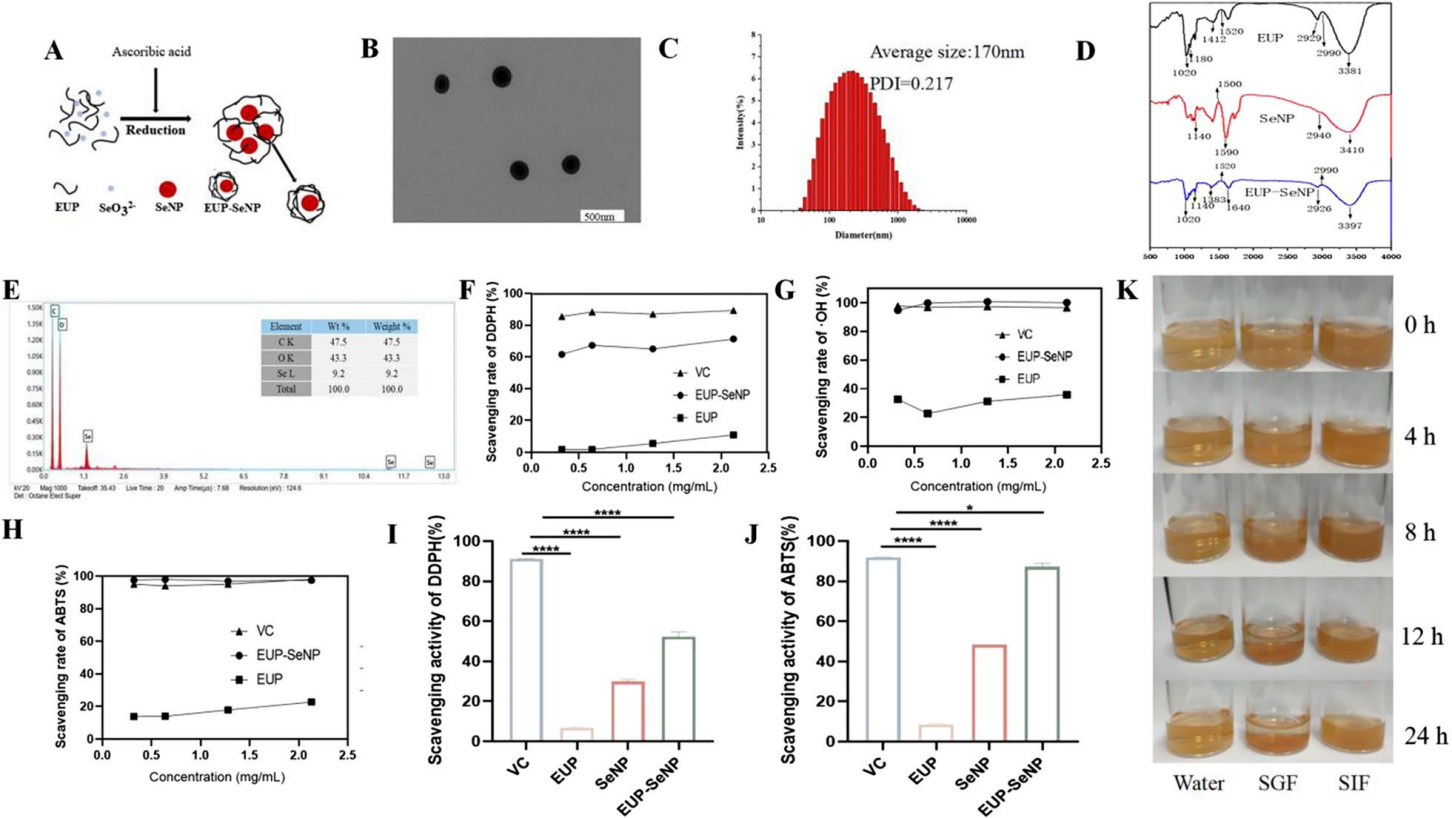
Preparation and characterization of EUP-SeNP. **A** Preparation process of EUP-SeNP; **B** TEM of EUP-SeNP; **C** Particle size of SeNPs prepared at different concentration of EUP; **D** FTIR spectra of EUP, SeNP and EUP-SeNP. **E** EDX spectrum; **F**–**H** DDPH,·OH and ABTS scavenging rate of EUP-SeNP modified with different concentrations of EUP. **I** DDPH scavenging rate of EUP, SeNP and EUP-SeNP. **J** ABTS scavenging rate of EUP, SeNP and EUP-SeNP. **K** The state of EUP-SeNP solution in SGF, SIF and water within 24 h

The preparation flow chart is shown in Fig. 1A. TEM image clearly showed a monodisperse and uniform spherical structure of EUP-SeNP (Fig. 1B). We selected different concentrations of EUP to modify SeNP, and found that 1.28 mg/mL EUP can control the particle size at 170 nm (Fig. 1C). After dialysis, EUP-SeNP particles were dried and analyzed by FTIR (Fig. 1D). EUP-SeNP showed characteristic peaks similar to EUP. The characteristic peak caused by hydroxyl stretching vibration shifted from 3381 cm^− 1^ to 3397 cm^− 1^. The C-O-C bond shifts from 1412 cm^− 1^ to 1383 cm^− 1^, which indicates that hydroxyl groups are adsorbed on the EUP-SeNP surface. In addition, the peak caused by C-H vibration also changed from 2929 cm^− 1^ to 2926 cm^− 1^, which further confirmed the combination of EUP and SeNP. Analysis of the elemental composition of EUP-SeNP using EDX (Fig. 1E) showed the presence of a strong signal from selenium atoms in EUP-SeNP. Subsequently, we explored the effect of EUP concentration on the antioxidant capacity of EUP-SeNP in vitro, and the results showed that the free radical scavenging rate of EUP-SeNP in vitro was independent of EUP concentration. The scavenging rates of DDPH, ·OH and ABTS free radicals were increased by 59.41%, 79.30% and 68.11% respectively by EUP-SeNP compared with EUP (Fig. 1F–H). In addition, we explored the free radical scavenging ability of 1.28 mg/mLEUP modified SeNP compared with SeNP alone, and found that he scavenging rates of DDPH and ABTS free radicals were increased by 57.34%, 55.41% respectively by EUP-SeNP compared with SeNP(Fig. 1I–J). Finally, we simulated the stability of EUP-SeNP in simulated gastroenteric fluid(SGF), simulated intestinal fluid(SIF) and water.(Fig. 1K). The results showed that EUP-SeNP solution was maintained for 4 h under the condition of SGF, and the homogeneous state did not accumulate. At subsequent points in time, small amounts of aggregates were observed, with the most aggregations occurring at 24 h, while EUP-SeNP remained homogeneous in water and SIF. These results indicate that SeNP modified with 1.28mg/mL EUP can obtain uniform and stable SeNP, it has a good free radical scavenging ability in vitro, and remains relatively stable in SGF, which is conducive to play a role in the intestine.

### Oral EUP-SeNPs alleviates DSS-induced colitis

**Fig. 2:**
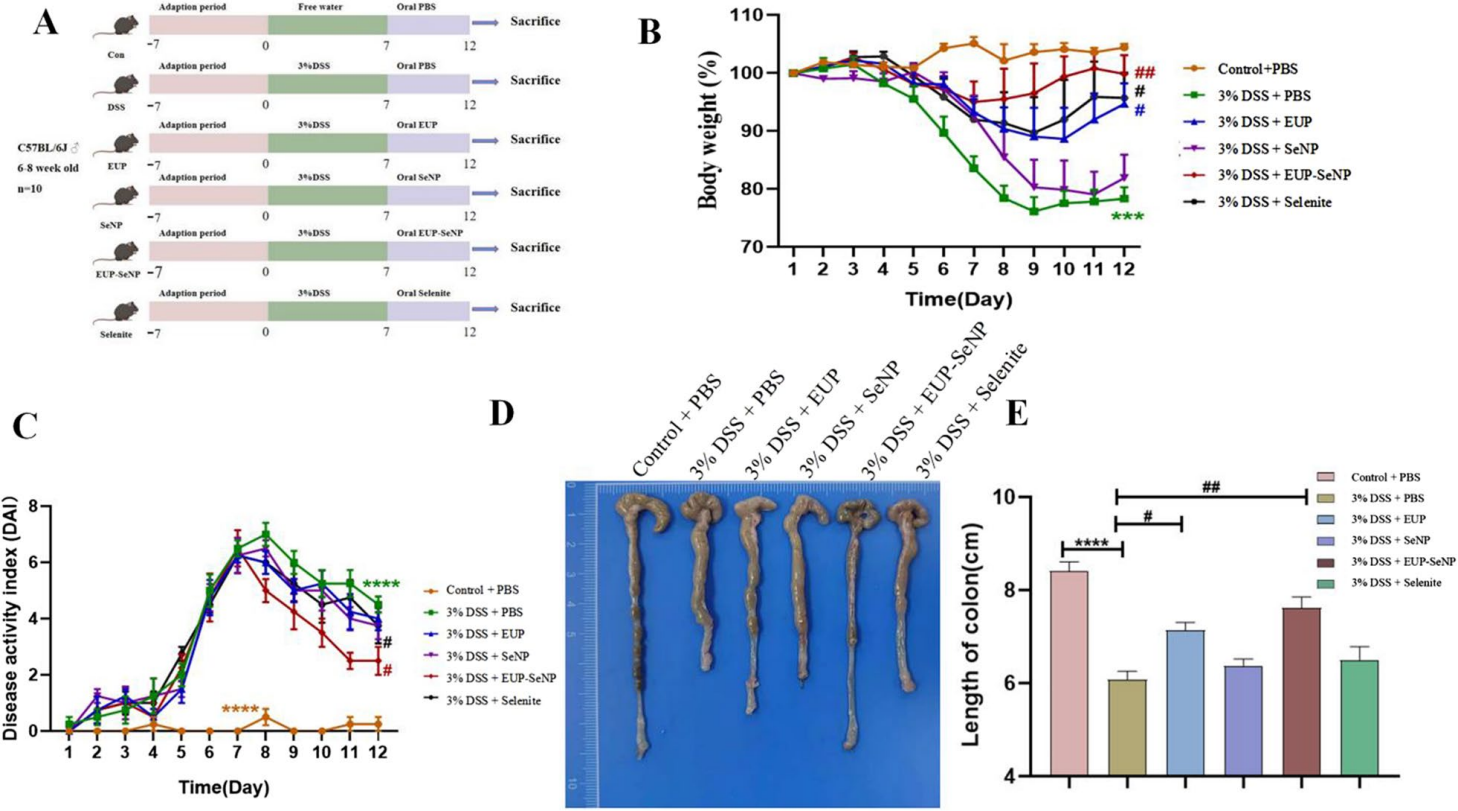
Oral EUP-SeNP improved the symptoms of DSS-induced colitis in mice. **A** Experimental treatment and grouping; **B** Weight loss (n = 10 for each group); **C** Disease activity index score changes; **D**, **E** colon length (n = 10). A dose of 3% w/v DSS drinking water was given for 7 days. Data are means ± SEM (n = 8 to 10 mice per group; “*”P < 0.05, “**”P < 0.01, “***”P < 0.001 and “****”P < 0.0001 by one-way ANOVA with Bonferroni’s multiple comparisons test. “#”P < 0.05, “##”P < 0.01, “###”P < 0.001 and “####”P < 0.0001 by one-way ANOVA with Bonferroni’s multiple comparisons test. “*” represents the significance between the control group and the DSS model group; “#” represents the significance between the DSS model group and the treatment group)

Experimental design as shown in Fig. 2A. The body weight of the mice decreased significantly at the end of 3% DSS modeling, after treatment with different substances for 5 days, the body weight of mice in the treatment group increased, and there was no significant difference between the oral EUP-SeNP treatment group and the control group (Fig. 2B). In addition, the results of DAI score indicated that on the 7th day of modeling, the DAI in the 3% DSS model group increased significantly compared with the control group (p < 0.0001). The oral EUP-SeNP treatment group significantly reduced the DAI score (p < 0.05) (Fig. 2C). Meanwhile, it can be also found that the colon length increased effectively in the oral EUP-SeNP treatment group compared to the model group (p < 0.01) (Fig. 2D–E). These results indicated that oral administration of EUP-SeNP could availably alleviate DSS-induced colitis.

### Oral EUP-SeNP improved DSS-induced intestinal permeability and increased antioxidant capacity

**Fig. 3:**
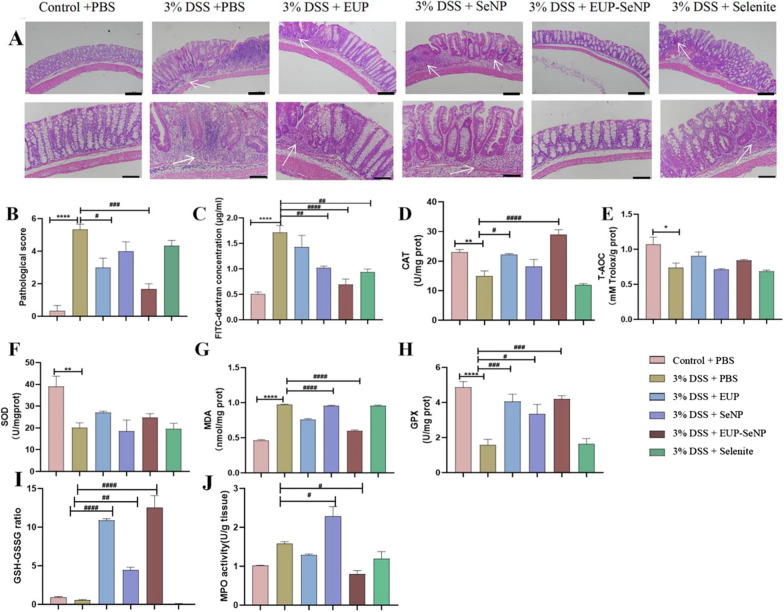
Oral EUP-SeNP improved DSS-induced intestinal permeability and increased antioxidant capacity. **A** H&E staining of colon with low and high power image, scale bar is 100 μm; **B** Pathological score of colon; **C** Changes in intestinal permeability. **D**–**J** The expression of CAT, T-AOC, SOD, MDA and GPX,GSH/GSSG and MPO activity of colon(n = 5). Data are means ± SEM by one-way ANOVA with Bonferroni’s multiple comparisons test). “*”P < 0.05, “**”P < 0.01, “***”P < 0.001 and “****”P < 0.0001 by one-way ANOVA with Bonferroni’s multiple comparisons test. “#”P < 0.05, “##”P < 0.01, “###”P < 0.001 and “####”P < 0.0001 by one-way ANOVA with Bonferroni’s multiple comparisons test. “*” represents the significance between the control group and the DSS model group; “#” represents the significance between the DSS model group and the treatment group

Low and high power images of colon H&E staining in different treatment groups (Fig. 3A). After 3% DSS free drinking, the colonic tissues of mice showed inflammatory cell infiltration and mucosal injury. Histopathological scores were significantly increased (Fig. 3B) and intestinal permeability was increased (Fig. 3C). All treatment groups could reduce the level of inflammation to some extent, particularly, after oral treatment with EUP-SeNP, the inflammatory cells of colon tissue were decreased, and the pathological score was significantly decreased compared with the model group(p < 0.001). Meanwhile, oral administration of EUP-SeNP effectively reduced the increased intestinal permeability caused by DSS(p < 0.0001). CAT, T-AOC, SOD, MDA, and GPX have been widely used as markers of oxidative stress. The levels of CAT, T-AOC, SOD and GPX in DSS-induced colitis mice decreased and the level of MDA increased (Fig. 3D–H). It is worth noting that the EUP-SeNP treatment group can significantly increase the levels of CAT(p < 0.0001) and GPX(p < 0.001) in colitis tissues, and also partly increase the levels of T-AOC and SOD. Furthermore, comparing with the model group, the level of MDA in colon tissue was significantly decreased in the EUP-SeNP treatment group(p < 0.0001). Additionally, oral EUP-SeNP significantly increased colon GSH/GSSH(p < 0.0001) (Fig. 3I) and reduced MPO level(p < 0.05) (Fig. 3J). These results manifested that EUP-SeNP can effectively alleviate DSS-induced colitis by reducing inflammatory cell infiltration and intestinal permeability, meanwhile improved the antioxidant capacity of colon tissue.

### Oral EUP-SeNP improved DSS-induced intestinal barrier function

**Fig. 4:**
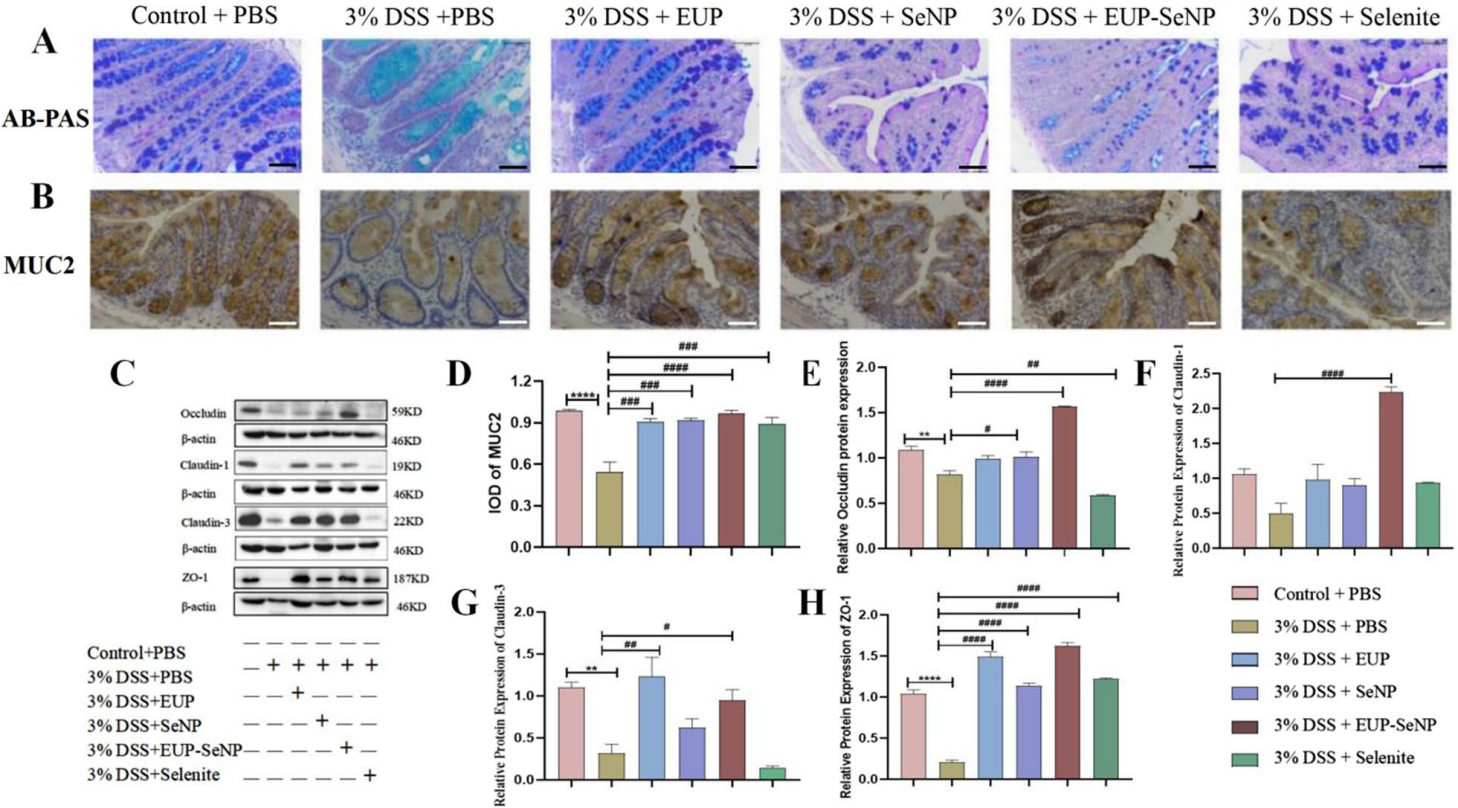
Oral EUP-SeNP improved DSS-induced intestinal barrier function. **A** AB-PAS staining of colon; the scale bar is 50 μm. **B** Mucin (MUC2) was stained by immunohistochemistry, the scale bar is 50 μm. **C** IOD was counted of MUC2 by immunohistochemical staining; **D** Protein bands analysis of colonic tight-junction proteins Occludin, claudin-1, claudin-3 and ZO-1; **E**–**H** Protein statistical analysis of colonic tight-junction proteins Occludin, claudin-1, claudin-3 and ZO-1. Data are means ± SEM by one-way ANOVA with Bonferroni’s multiple comparisons test. “*”P < 0.05, “**”P < 0.01, “***”P < 0.001 and “****”P < 0.0001 by one-way ANOVA with Bonferroni’s multiple comparisons test. “#”P < 0.05, “##”P < 0.01, “###”P < 0.001 and “####”P < 0.0001 by one-way ANOVA with Bonferroni’s multiple comparisons test. “*” represents the significance between the control group and the DSS model group; “#” represents the significance between the DSS model group and the treatment group

DSS-induced colitis is accompanied by a decrease in the number of goblet cells and a decrease in mucin, which was ameliorated by increasing the number of goblet cells and mucin secretion in the treatment group compared with the DSS group (Fig. 4A–B). The expression of tight junction protein of colon is shown in Fig. 4C. We found that the EUP-SeNP treatment group significantly increased the expression of MUC2 in colon tissues compared with the model group(p < 0.001) (Fig. 4D). EUP-SeNP treatment group can effectively improve intestinal barrier damage caused by DSS, which is manifested in significantly increased expression of tight Occludin(p < 0.001), Claudin-1(p < 0.001), Claudin-3(p < 0.05) and ZO-1(p < 0.001), comparing with DSS model group, respectively. It should be noted that other treatment groups can also improve the expression of tight junction protein, but the therapeutic effect is not as good as that of EUP-SeNP (Fig. 4E–H).

### Oral EUP-SeNP alleviated DSS-induced colitis by improving level of apoptosis, proliferation and inflammatory

**Fig. 5:**
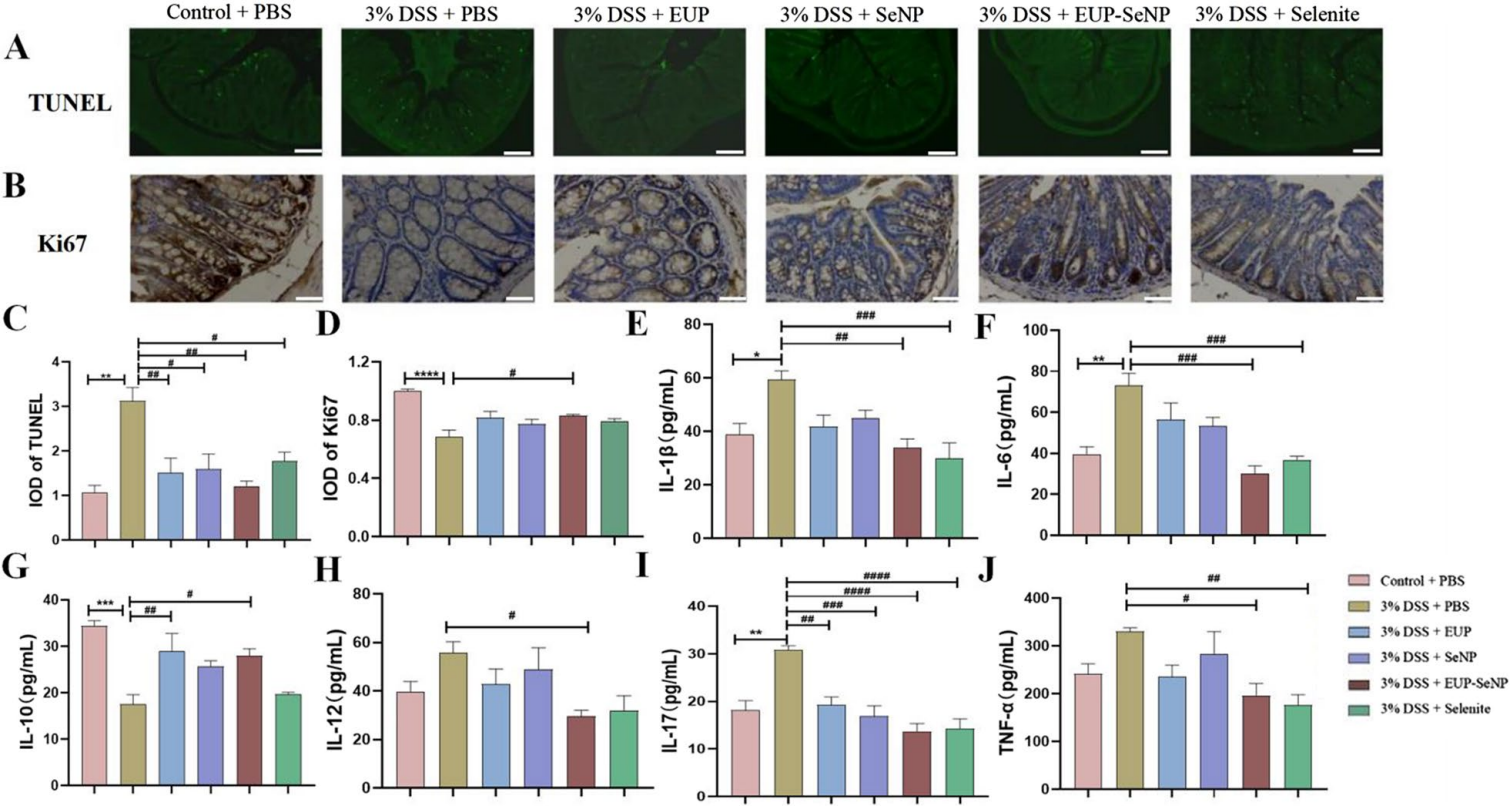
Oral EUP-SeNP alleviated DSS-induced colitis by improving level of apoptosis, proliferation and inflammatory. **A **TUNEL staining of colon, the scale bar is 50 μm. **B** Ki67 was stained by immunohistochemistry, the scale bar is 50 μm. **C** IOD was counted of TUNEL by Immunofluorescence staining; **D** IOD was counted of Ki67 by immunohistochemical staining. **E**–**J** The expression of IL-1β, IL-6, IL-10, IL-12, IL-17 and TNF-α proteins in colon was detected by ELISA. Data are means ± SEM by one-way ANOVA with Bonferroni’s multiple comparisons test. “*”P < 0.05, “**”P < 0.01, “***”P < 0.001 and “****”P < 0.0001 by one-way ANOVA with Bonferroni’s multiple comparisons test. “#”P < 0.05, “##”P < 0.01, “###”P < 0.001 and “####”P < 0.0001 by one-way ANOVA with Bonferroni’s multiple comparisons test. “*” represents the significance between the control group and the DSS model group; “#” represents the significance between the DSS model group and the treatment group

TUNEL and Ki67 staining were used to measure the number of apoptotic cells and epithelial cell proliferation, respectively (Fig. 5A–B). The results showed that DSS resulted in increased apoptosis (Fig. 5C) and decreased proliferation (Fig. 5D) of colonic epithelial cells, EUP-SeNP treatment group can significantly reduce the level of apoptosis cell (p < 0.01) and improve the proliferation cell of colon tissue (p < 0.05). Subsequently, we further analyzed the level of inflammatory factors in colon tissue and found that the pro-inflammatory cytokines IL-1β, IL-6, IL-12, IL-17 and TNF-α were increased and the anti-inflammatory cytokine IL-10 was decreased in the colon of DSS-induced colitis (Fig. 5E–J). However, Different treatment groups showed the potential of regulating cytokine levels, in which EUP-SENP could significantly increase the expression of IL-10(p < 0.05) and decrease the expression of IL-1β(p < 0.01), IL-6(p < 0.001), IL-12(p < 0.05), IL-17(p < 0.001) and TNF-α(p < 0.05). These results indicated that oral administration of EUP-SeNP could ameliorate DSS-induced colonic injury by regulating the apoptosis and proliferation of intestinal epithelial cells and the expression of inflammatory cytokines.

### Oral EUP-SeNP regulated intestinal microbiota composition

**Fig. 6:**
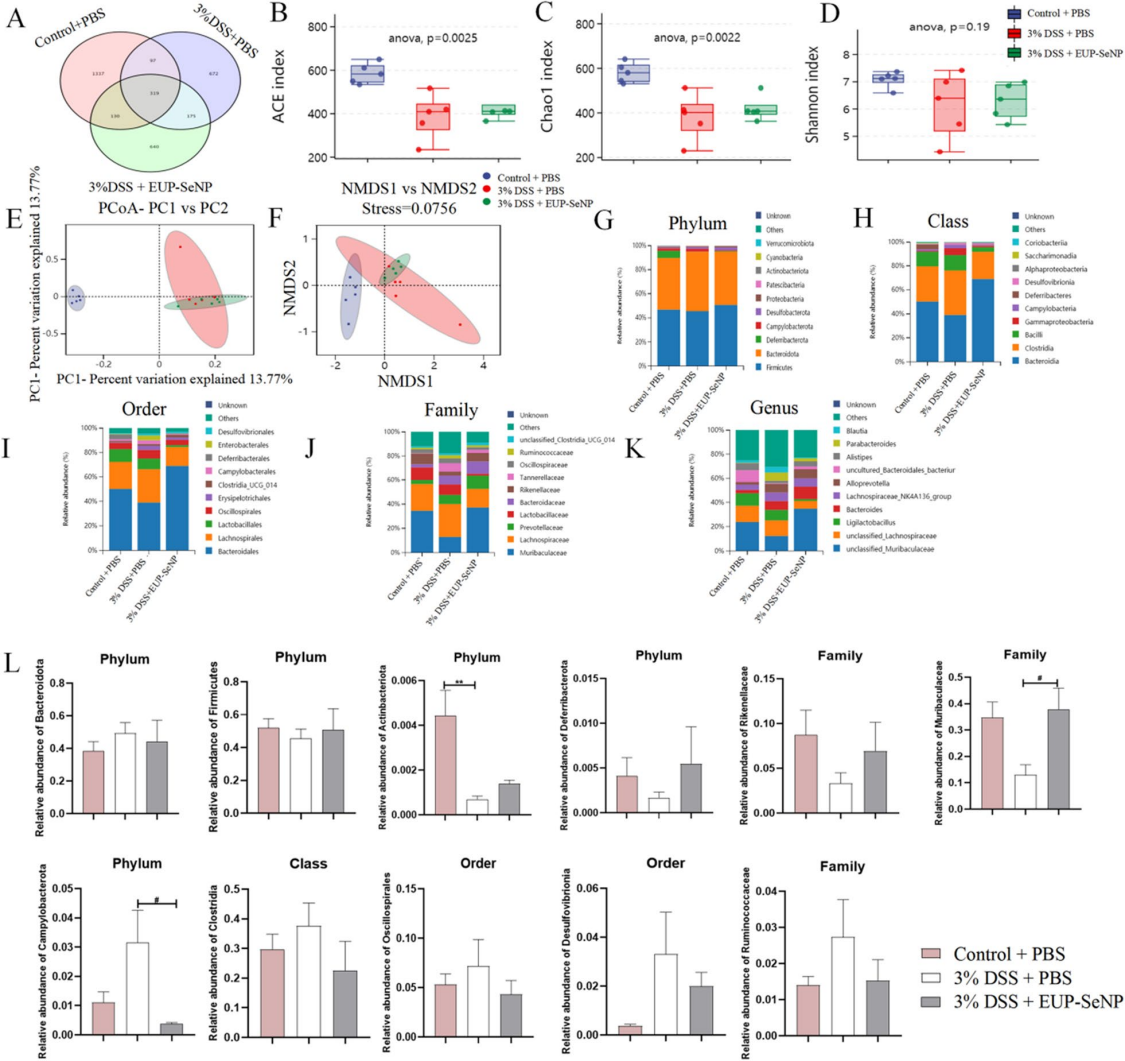
Oral EUP-SeNP altered the microbial composition of the colon in DSS-treated mice. **A** Veen diagram of colonic microorganisms OTUs. **B** ACE index. **C** Chao1 index. **D** Shannon index. **E** Principal coordinates analysis (PCoA) of colonic microorganisms. **F** Non-metric multi-dimensional scaling (NMDS) of colonic microorganisms. **G**–**K** Colon microbiota composition at Phlyum, Class, Order, Family and Genus level. **L** Changes in the abundance of some beneficial and harmful bacteria in colon tissue. Data are means ± SEM. (n = 5) “*”P < 0.05, “**”P < 0.01, “***”P < 0.001 and “****”P < 0.0001 by one-way ANOVA with Bonferroni’s multiple comparisons test. “#”P < 0.05, “##”P < 0.01, “###”P < 0.001 and “####”P < 0.0001 by one-way ANOVA with Bonferroni’s multiple comparisons test. “*” represents the significance between the control group and the DSS model group; “#” represents the significance between the DSS model group and the treatment group

16 S rRNA gene sequencing was used to detect the colonic microbiota of mice in control, DSS and EUP-SeNP treatment group. OTUs Veen diagram showed that there were 319 common OTUs in the colonic microbiota of mice in each group (Fig. 6A). ACE, Chao1 index and Shannon index have changed to some extent (Fig. 6B–D). There was no significant change in α diversity, which may be related to the time of modeling. Principal Coordinate analysis (PCoA) (Fig. 6E) and Non-metric multidimensional scaling (NMDS)(Fig. 6F) showed that compared with the DSS model group, the intestinal microbiota composition of the EUP-SeNP treatment group and the control group were more similar and closer together. We analyzed the significance of differences between groups at different classification levels (Fig. 6G–K), and the results showed that the composition of intestinal microbiota changed after DSS induced mice. DSS model group increased the abundance of Bacteroidetes and decreased the abundance of Firmicutes. In addition, the abundance of beneficial bacteria in colon tissues, such as Actinobacteriota, deferribacterota, Rikenellaceae, and Muribaculaceae, decreased after drinking water containing 3% DSS. On the contrary, The abundance of harmful bacteria such as campylobacterota, colstridia, oscillospirales, Desulfovibria and Ruminococcaceae increased (Fig. 6L). In conclusion, oral administration of EUP-SeNP can regulate the intestinal microbiota composition by increasing the abundance of beneficial flora and decreasing the abundance of harmful flora.

### Oral EUP-SeNP does not cause damage to the body

**Fig. 7:**
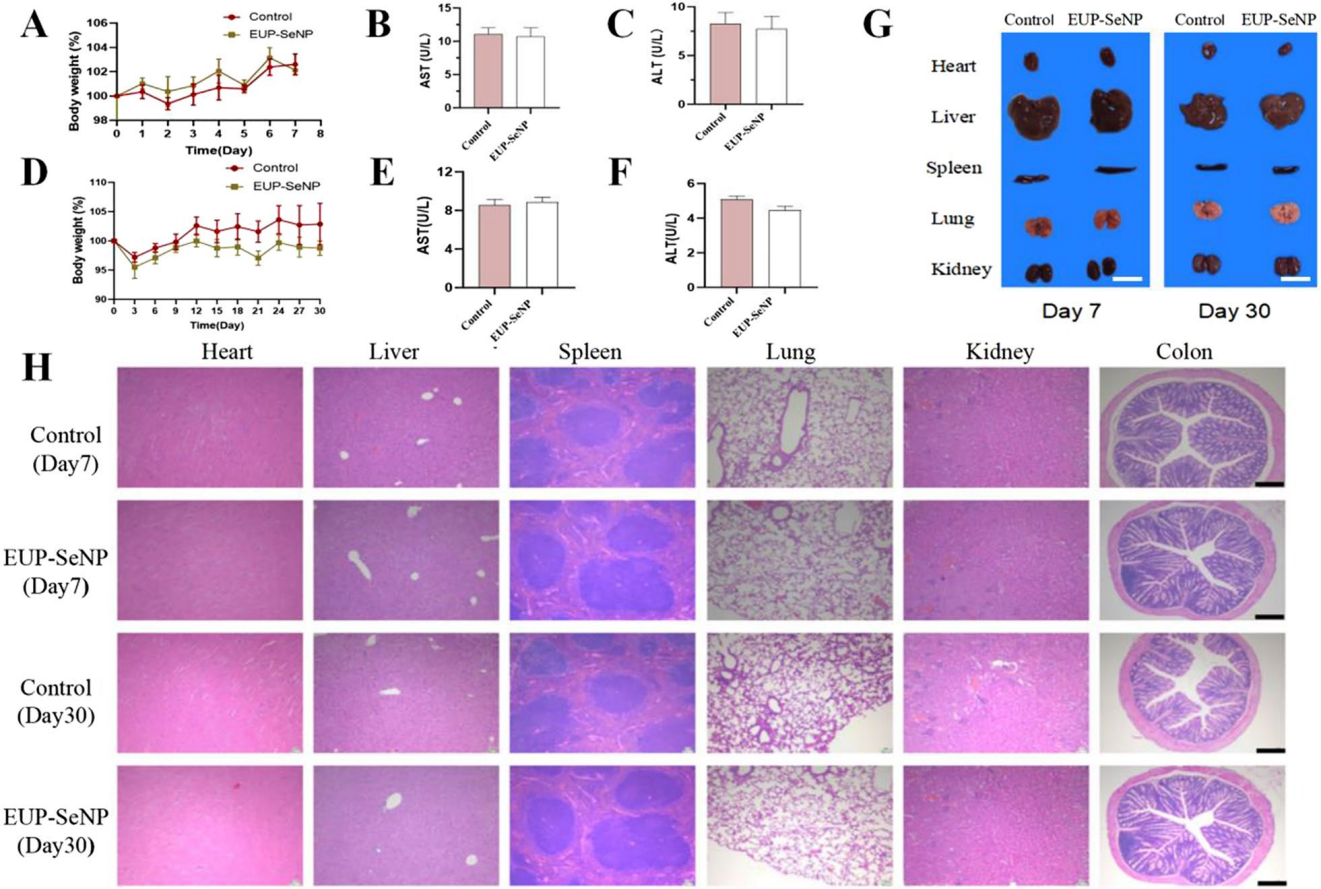
Oral EUP-SeNP does not cause damage to body. We simulated the short-term and long-term toxic effects of EUP-SeNP, administered orally for 7 days and 30 days. **A** Seven-day weight change (n = 5); **B** Oral administration of EUP-SeNP serum AST for 7 days; **C** Oral administration of EUP-SeNP serum ALT for 7 days; **D** Thirty-day weight change (n = 5); **E** Oral administration of EUP-SeNP serum AST for thirty days; **F** Oral administration of EUP-SeNP serum ALT for 30 days; **G** Morphology of organs at necropsy, scale bar is 1 cm. **H** H&E staining of heart, liver, spleen, lung, kidney and colon after 7 and 30 days of oral EUP-SeNP, scale bar is 100 μm

In the control group, there was no significant change in body weight (Fig. 7A) and no difference in AST(Fig. 7B) and ALT (Fig. 7C) compared with that in the oral administration of EUP-SeNP for 7 days. In addition, we also tested the effects of oral EUP-SeNP on body weight (Fig. 7D), AST (Fig. 7E) and ALT(Fig. 7F) after 30 days, the results showed that there were no significant changes between the control and oral EUP-SeNP group. In addition, the appearance of each organ was observed without abnormalities (Fig. 7G). Finally, we performed H&E staining on each organ (Fig. 7H), and the results manifested that there was no difference between the control group and the oral EUP-SeNP group. These results revealed that oral administration of EUP-SeNP has no toxic effect on the body in the short and long term and it has good biocompatibility.

### EUP-SeNP is nontoxic to IEC-6 at certain doses and can be taken up by cells

**Fig. 8:**
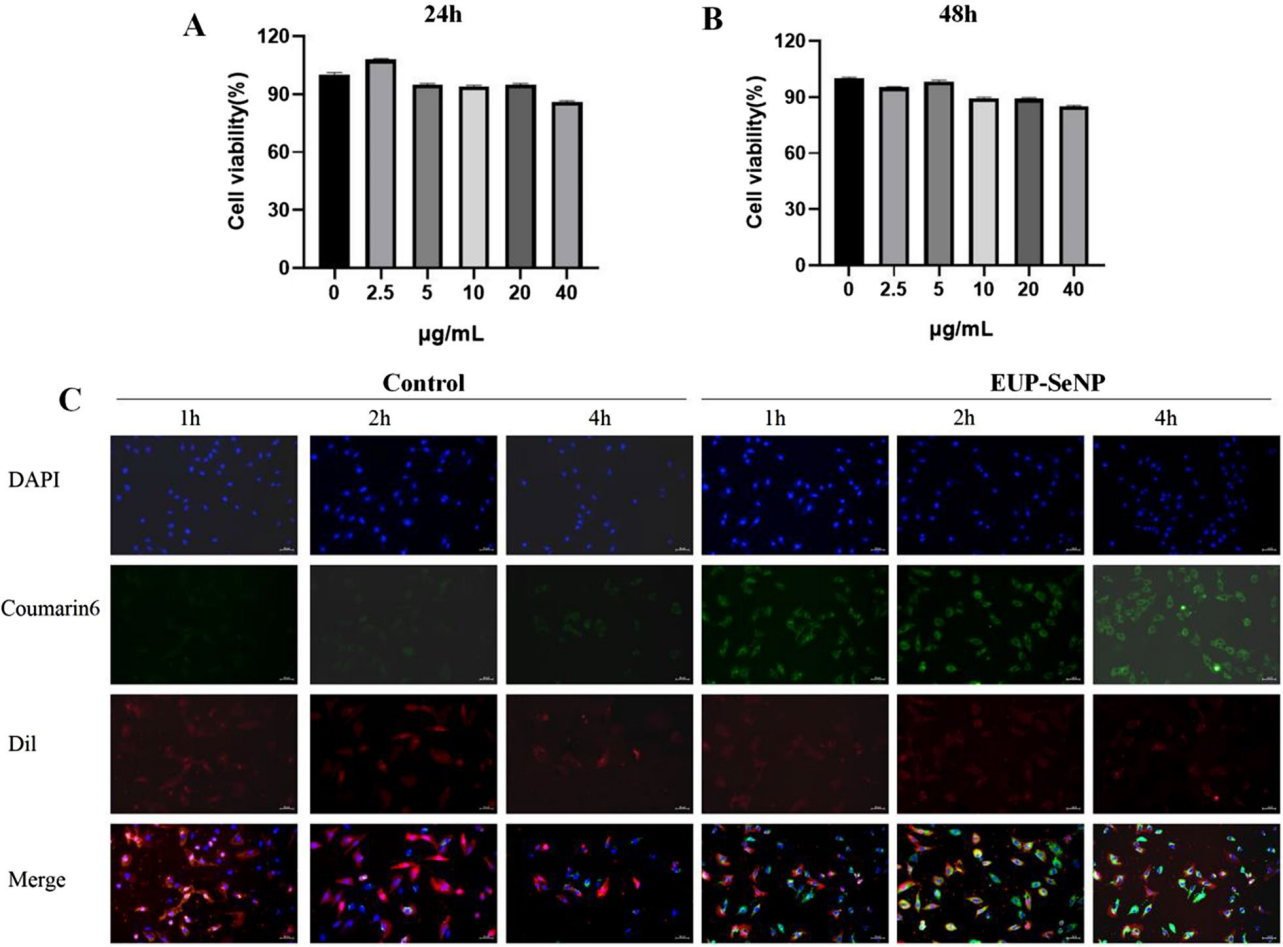
EUP-SeNP is nontoxic to IEC-6 at certain doses and can be taken up by cells. **A** Cell viability of IEC-6 treated with different concentrations of EUP-SeNP for 24 h. **B** Cell viability of IEC-6 treated with different concentrations of EUP-SeNP for 48 h. **C** Uptake of coumarin6-labeled EUP-SeNP by IEC-6 at different times, the concentration of EUP-SeNP was 10 µg/mL, scale bar is 50 μm

The different concentrations of EUP-SeNP did not affect the activity of IEC-6 within 24 and 48 h (Fig. 8A–B). Subsequently, Cellular uptake of EUP-SeNP by IEC-6 at different time points showed that EUP-SeNP uptake by IEC-6 was time-dependent, with most EUP-SeNP uptake by IEC-6 cells at 4 h. Simultaneously, Dil was also used to stain cell membranes. Obviously, EUP-SeNP is localized in the cytoplasm rather than the cell surface (Fig. 8C).

### EUP-SeNP protects against LPS-induced cell injury by enhancing tight junction protein expression and inhibiting TRL-4/NF-κB signaling pathway activation.

**Fig. 9:**
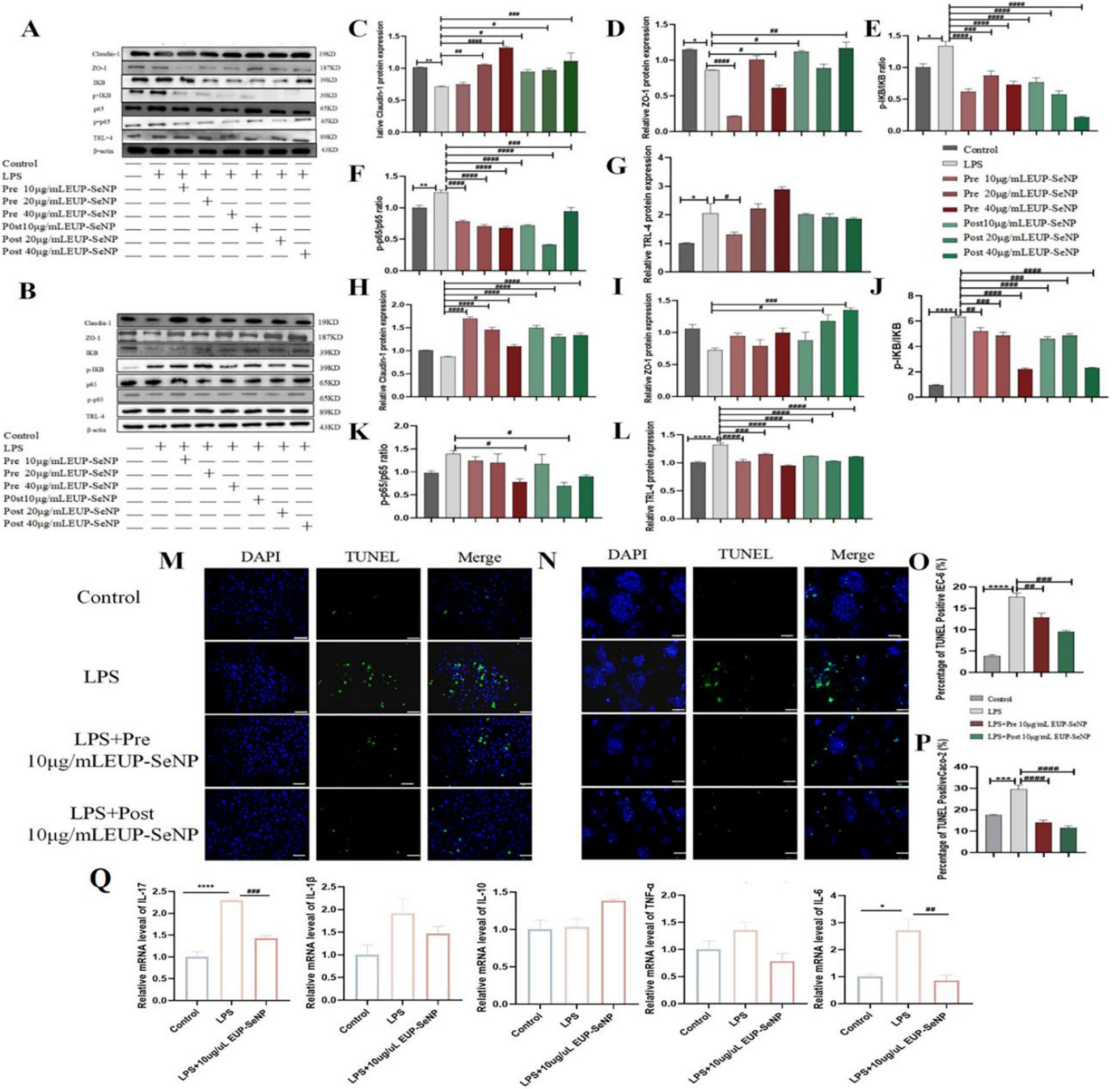
EUP-SeNP can protect against LPS-induced cell injury by enhancing intestinal barrier function and regulating NF-KB signaling pathway. **A** Protein bands analysis of IEC-6; **B** Protein bands analysis of Caco-2; **E**–**G** Protein statistical analysis of claudin-1, ZO-1, pIKB/IKB, pp65/p65 and TRL-4 of IEC-6. **H**, **I** Protein statistical analysis of claudin-1,ZO-1, pIKB/IKB, pp65/p65 and TRL-4 of Caco-2. **M** TUNEL staining of IEC-6, the scale bar is 50 μm. **N** TUNEL staining of Caco-2, the scale bar is 50 μm. IEC-6 and Caco-2 were induced by LPS for 36 h, and the Pre and Post groups were treated with 10ug/mL EUP-SeNP for 12 h. **O** IOD of TUNEL staining ofEC-6. **P** IOD of TUNEL staining of Caco-2. **Q** mRNA expression of inflammatory cytokines IL-17,IL-1β, IL-10,TNF-α, and IL-6 in IEC-6. Data are means ± SEM by one-way ANOVA with Bonferroni’s multiple comparisons test. “*”P < 0.05, “**”P < 0.01, “***”P < 0.001 and “****”P < 0.0001 by one-way ANOVA with Bonferroni’s multiple comparisons test. “#”P < 0.05, “##”P < 0.01, “###”P < 0.001 and “####”P < 0.0001 by one-way ANOVA with Bonferroni’s multiple comparisons test. “*” represents the significance between the control group and the DSS model group; “#” represents the significance between the DSS model group and the treatment group

IEC-6 protein expression (Fig. 9A) and Caco-2 protein expression (Fig. 9B) were detected after LPS induction. The statistical results were exhibited in Fig. 9C–G and H-L, respectively. We found that the expression of tight junction proteins claudin-1 (Fig. 9C and H) and ZO-1 (Fig. 9D and I) of IEC-6 and Caco-2 decreased after LPS introduction. After treatment with different concentrations of EUP-SeNP, tight junction protein expression was increased in both prevention and post groups. Additionally, We detected the expression of p-IκB/IκB (Fig. 9E, J), pp65/p65 (Fig. 9F and H) and TRL-4 (Fig. 9G, L) of IEC-6 and Caco-2, the results showed that LPS can activate the TRL-4/NF-κB signaling pathway and increase the level of these protein. However, different concentrations of EUP-SeNP could inhibit this signaling pathway and decrease these protein expression in both prevention and post-treatment groups. TUNEL staining was used to explore the effect of LPS on IEC-6 (Fig. 9M) and Caco-2 (Fig. 9N) apoptotic cells. TUNEL-positive cells were reduced in both prevention and post-treatment groups, observably. Subsequently, we explored whether EUP-SeNP could resist the damage of LPS to IEC-6 by regulating inflammation. The results showed that EUP-SeNP significantly reduced mRNA expression of IL-17(p < 0.001) and IL-6, correspondingly decreased IL-1β and TNF-α, and increased the expression of IL-10(P < 0.01) (Fig. 9Q). These results indicated that EUP-SeNP could enhance the expression of tight junction protein, inhibit the activation of TRL-4/NF-κB signaling pathway and reduce the number of apoptotic cells and regulate inflammatory levels to alleviate the adverse effects of LPS on cells.

## Discussion

Due to the complex and diverse causes of UC, the current treatment is troublesome [2, 28, 29]. Numerous studies on UC have shown that the current treatment can only play a palliative role, such as reducing the level of inflammation, regulating the composition of intestinal flora, enhancing intestinal barrier function and so on [30, 31]. With the high incidence of UC, in addition to the use of aminosalicylic acid, adrenocortical hormones, immunosuppressants, biologics and other drugs [32–34], researchers are constantly exploring new therapies.

Many years ago, Se was widely considered to be biologically inert, but recently, comparing with other forms of Se [9], researchers have demonstrated that SeNP in the zero-valent state can not only effectively up-regulate selenase, but also have a lower toxicity [14]. Red SeNP prepared by chemical method is easy to precipitate and tend to aggregate into black particles due to lack of terminal agent. Therefore, proteins, polysaccharides, polyphenols and other biological macromolecules are often used to solve this shortcoming of SeNP [18, 21, 22].

DSS induced mouse colitis model has been widely used to study animal models of UC because of its similar clinical symptoms to ulcerative colitis [35, 36]. After induction of DSS, intestinal barrier was destroyed, microflora was disturbed and intestinal function was abnormal in mice [28, 37]. We investigated the effect of EUP-SeNP on DSS induced colitis in mice from the aspects of mucosal barrier, inflammation level and microbial composition. Mucosal mechanical barrier and intestinal mucosal microbial barrier are essential for maintaining intestinal function. Mucin in the mucous layer can availably prevent the invasion of pathogens and microorganisms [38, 39]. Tight junction protein is located at the top of the side membrane surface of intestinal mucosal epithelial cells, forming the structural basis of intestinal barrier function [40]. After DSS induction, the levels of mucin and tight junction protein were decreased. Surprisingly, the levels of mucin and tight junction protein were significantly increased in the Oral EUP-SeNP treatment group compared with the model group. Inflammatory response is an important indicator of the inflammatory state of the body [41]. We evaluated the cytokine levels in colon tissue, and found that the anti-inflammatory cytokine IL-10 was significantly increased after oral EUP-SeNP treatment, while the pro-inflammatory cytokine IL-1β, IL-6, IL-12, IL-17, and TNF-α were decreased compared with the model group. Redox homeostasis is an essential factor in maintaining the homeostasis of the body. There are various indicators of colon redox state, and GSH/GSSG is an important indicator used to evaluate the body’s redox state. When redox state is in disorder, the ratio decreases [42]. In colitis mice, colon tissue MPO activity was used to detect neutrophil infiltration into the inflamed colon mucosa. T-AOC, CAT, GPX, SOD and MDA were also used to detect tissue antioxidants [6, 43]. Our results suggested that oral EUP-SeNP can effectively improve colon antioxidant capacity and alleviate the severity of DSS induced colitis.

Subsequently, We analyzed the colon microbiome and it was noteworthy that although there was no difference in α-diversity analysis among groups, PCoA and NMDS results showed changes in intestinal microbiome composition among groups. And we suspected that this might be due only to the timing of the mold, which also has been seen in previous studies [44]. Our study found apparent changes in the composition of the flora at different taxonomic levels, which may also exacerbate the severity of colitis. In oral EUP-SeNP treatment group, the composition of colon microbiome can be adjusted, specifically as follows, Oral EUP-SeNP treatment group can effectively increase the decrease in the abundance of beneficial bacteria caused by DSS, such as Actinobacteriota, deferribacterota, Rikenellaceae, and Muribaculaceae. It can also reduce the abundance of harmful bacteria such as Campylobacterota, Colstridia, Oscillospirales, Desulfovibria and Ruminococcaceae. These results are also consistent with previous studies [35, 45-47].

Finally, we verified the mechanism of action of EUP-SeNP at the cellular level. LPS is a good inducer of cellular inflammatory models. LPS can destroy intestinal mucosal barrier and reduce the expression of tight junction protein [48, 49]. Therefore, we detected tight junction protein expression in IEC-6 and Caco-2, and found that LPS-induced reduced tight junction protein expression, while EUP-SeNP treatment enhanced tight junction protein expression. In addition, since NF-κB also plays a key role in inflammatory responses and is usually inactive in the cytoplasm, it is phosphorylated in response to extracellular factor stimulation. Phosphorylated NF-κB then enters the nucleus, binds to target genes and regulates transcription [50, 51]. TLR-4, as a pattern recognition receptor, plays a key role in intestinal anti-pathogen defense. And TLR4-mediated inflammatory related intestinal damage can accelerate the development of UC [52, 53]. Inhibition of TLR4/NF-κB signaling pathway is a crucial mechanism for the action of many anti-inflammatory drugs [54]. We proved LPS-induced activation of the TRL-4/NF-κB signaling pathway, and EUP-SeNP treatment can effectively inhibit the activation of this pathway and reduce the level of inflammation. In conclusion, we successfully constructed a stable EUP-SeNP with good antioxidant capacity in vitro for the first time, which can effectively reduce DSS induced colitis in mice and inhibit the activation of the cellular TRL-4/NF-κB pathway. We speculate that EUP-SeNP may have certain potential in the clinical treatment of IBD.

## Conclusion

We prepared a uniform spherical shape EUP-SeNP with a particle size of about 170 nm, which has certain antioxidant function in vitro and in vivo. It can reduce DSS-induced colitis by enhancing intestinal mucosal barrier function, reducing inflammation level, improving antioxidant function, promoting proliferation and reducing apoptotic cells. EUP-SeNP can also regulate the composition of intestinal flora, increase the abundance of beneficial bacteria, reduce the abundance of harmful bacteria and improve colon injury. In addition, EUP-SeNP protects against LPS-induced cell damage by inhibiting the activation of TRL-4/NF-κB signaling.

## Data Availability

All data and materials during this study are included in this manuscript.

## Abbreviations

AB-PAS: Alcian blue-periodic acid-Schiff
ABTS: 2, 2’-azino-bis(3-ethylbenzothiazoline-6-sulfonic acid)
CAT: Catalase
DAI: 1,1-diphenyl-2-picrylhydrazyl
DDPH: 1,1-diphenyl-2-picrylhydrazyl
DSS: Glucan sodium sulfate
EDX: Energy dispersive X-ray spectroscopy
EUP: *Eucommia* ulmoides polysaccharide
EUP-SeNP: *Eucommia* ulmoides polysaccharide-nano-selenium particle
FTIR: Fourier transform infrared
GPX: Glutathione peroxidase
GSH/GSSG: Glutathione/oxidized Glutathione
H&E: Hematoxylin&eosin
IBD: Inflammatory bowel disease
LPS: Lipopolysaccharide
MDA: Malondialdehyde
MPO: Myeloperoxidase
NMDS: Non-metric multi-dimensional scaling
OH: Hydroxyl radical
PCA: Principal component analysis
PCoA: principal coordinates analysis
Se: Selenium
SeNP: Nano-selenium particle
SGF: Simulated gastroenteric fluid
SIF: Simulated intestinal fluid
SOD: Superoxide Dismutase
T-AOC: Total antioxidant capacity
TEM: Transmission electron microscopy
UC: Ulcerative colitis
